# Health-Related Challenges and Programs Among Agriculture Workers: A Narrative Review

**DOI:** 10.7759/cureus.57222

**Published:** 2024-03-29

**Authors:** Dipali Khode, Ankita Hepat, Abhay Mudey, Abhishek Joshi

**Affiliations:** 1 Department of Community Medicine, Jawaharlal Nehru Medical College, School of Epidemiology and Public Health, Datta Meghe Institute of Higher Education and Research, Wardha, IND

**Keywords:** occupational hazard, morbidity, pesticide used, personal protective equipment, agricultural workers

## Abstract

Agriculture is one of the most hazardous occupations, with many workers experiencing occupational accidents and ill health. The misuse of toxic substances, often due to inadequate protective measures, raises concerns about both individual and nature safety. However, the strenuous tasks done by the agricultural workers, especially those related to pesticide exposure and some challenges affect the farmer's health and well-being. This review paper used databases like PubMed and Google Scholar to elaborate this. English language studies are included and other languages are excluded. The health system for agricultural labour in India sheds light on the neglected status of agricultural workers and emphasizes the need for health promotion programs. For that, training and interventions are important as crucial elements in reducing pesticide exposure, with a call for the enforcement of existing laws and regulations. Agriculture workers have a proper knowledge and attitude towards the safety and program to overcome the health-related conditions they face. This paper also addresses the practices of personal protective equipment (PPE) and the challenges faced by farmers in adopting adequate safety measures.

## Introduction and background

The World Health Organization (WHO) Alma Ata declaration in 1978 marked the origin of Basic Occupational Health Services (BOHS). The declaration stated that primary healthcare is vital, and it should be based on possible, scientifically sound, and socially acceptable methods. It should be easily accessible to individuals, families, and communities and should be brought nearby able to be done where individuals live and work [1]. Agricultural laborer is defined as "The people who are engaged in raising crops on payment of wages” [2]. The combination of high humidity, extreme ambient temperatures, intense physical labor, and insufficient fluid intake puts individuals at significant risk for heat strain and dehydration. These are serious hazards in the agricultural sector globally. Agricultural workers perform physically demanding tasks and are exposed to several health hazards and risks [3]. Based on research findings and information from public health and disease ecology, it was believed that the development of agricultural economies would inevitably result in increased morbidity and mortality rates, as well as a range of societal issues [4].

The growing global population demands food production for an estimated 9.1 billion people by 2050 [5]. Workers in the agriculture industry face a high risk of illness and injury. In 2017, agricultural crop workers reported 5.2 worktime injuries per 100 workers and experienced 20.9 deaths per 100,000 workers. Pesticide poisoning represents a major global public health concern, with up to 300,000 deaths attributed to it annually worldwide [5,6]. Every year, between 500,000 and 1 million people worldwide experience health problems due to pesticide poisoning, according to the World Health Organization [7]. In low and middle-income countries like India and Nepal in Asia, there has been a gradual decline in the yearly consumption of food calories, ranging from 0.8% to 2.2% annually [8]. In terms of farm outputs, India ranks second globally. According to the Indian Economic Survey 2020-21, more than 50% of the Indian manpower is employed in farming, and 20.2% of the country's gross domestic product (GDP) is contributed by it. India has the greatest net cropped area in the world, followed by the US and China [2]. With the introduction of the Green Revolution, a program aimed at enhancing global agriculture production, the use of pesticides in modern farming has become widespread [6]. As high as 19.4% of China's farmed land has heavy metal concentrations beyond permissible limits, according to the National Soil Pollution Survey Bulletin [9]. However, the misuse of pesticides is a serious concern in developing countries due to potential harm to personal and environmental safety [10]. This is crucial given current zoonotic research that predicts climate changes and further socio-ecological drivers will probably worsen the current illness burden, especially in developing countries like India with poor health structures [11]. This review aims to Identify the diverse array of health-related problems agricultural workers encounter, including physical, mental, and infectious disease-related challenges, and also understand the impact of hazards on agriculture workers [8].

The Worker Protection Standard (WPS) was first published by the Environmental Protection Agency (EPA) in 1974. The standard applies to those who perform manual labor after pesticide applications [12]. Farmers, especially those directly handling toxicants, have a higher risk of pesticide exposure. The risk of heat strain and dehydration is further exacerbated when individuals come into contact with pesticide residues on crops and engage in improper handling and storage practices [13]. The issue is not the lack of personal protective equipment (PPE) usage rather, agricultural workers use protective devices inadequately [14]. The education level plays a crucial role in enhancing awareness about the risks associated with pesticides [15]. Recently, innovative and strategic international partnerships aiming for sustainable agriculture in Africa have emerged. Their goal is to increase 30 million smallholder farming households' income and food security within five agricultural hotspots [16]. Fortunately, policymakers and developers acknowledge the necessity of supporting farmers in urban fringe regions. This support can resilience of the food mechanism and efficiently manage row materials while absorbing pressures on the framework [8].

## Review

Methodology

This review paper discusses health-related challenges and programs among agriculture workers. PubMed, Google Scholar, and Scopus were searched for peer-reviewed articles. The relevant English language, free full text articles included in the review, range from articles is 2013 to 2023. For this review, the Medical Subject Heading (MeSH), is used as ‘Agriculture Worker,’ ‘Occupational Health,’ and ‘Personal Protective equipment.’ Some filters used to find relevant data for the review are (agriculture worker) and (mortality [Title/Abstract]) agriculture worker [Title/Abstract] AND (personal protective equipment [Title/Abstract]) (agriculture worker [MeSH Terms]) AND occupational health [MeSH Terms]). Furthermore, details used some keywords like personal protective equipment, morbidity, agriculture workers, health safety, and occupational health. 

Health problems faced by agricultural labor

The use of pesticides carries the risk of harmful health effects due to which workers may be exposed to physical, chemical, and biological health hazards [17,18]. The most commonly used pesticide is glyphosate its formulations are hazardous to human cells when used in vitro and have been linked to a variety of cancers [19]. Also, inadequate information and training can pose serious health risks for farmers [20]. According to recent research, farmers commonly experience work-related skin irritation, back pain, headaches, dizziness, visual problems, and respiratory difficulties after spraying pesticides [21]. Musculoskeletal pain is usual in mature farmers. Pain in the back and lower limbs increases due to overwork, while pain in the upper extremities is less common [22]. The observed excess incidence of multiple myeloma in men in agricultural settings suggests the existence of both preventive and risk factors for cancer [23]. Pesticides have the potential to enter the human body through skin absorption, inhalation, and, to a lesser extent, ingestion. Acute intoxication is the result of significant exposure to a particular chemical over a short period. On the other hand, long-term exposure to hazardous substances or various products can lead to chronic intoxication, resulting in irreversible damage such as paralysis and neoplasia [6]. Seventy-seven percent of the farmers used hand pumps to spray pesticides, while the remaining 23% used their hands [7]. The study suggests that farm workers who were exposed to toxic substances have higher levels of biomarkers due to unsafe handling practices and insufficient risk perception [19]. These chemicals can have various harmful effects, including carcinogenesis, neurotoxicity, reproductive problems, and immunological effects. Some of these effects may only be apparent after 18 years of exposure and are poorly understood [6].

Musculoskeletal Disorders

Agricultural workers commonly experience musculoskeletal disorders, including back, upper limb, and lower limb pain, due to repetitive movements, awkward or stretched postures, and standing. The prevalence of musculoskeletal disorders in India is significant, with 52% of farmers reporting low back pain [22].

Pesticide Poising

In the agriculture sector, pesticides are commonly used, without the correct PPE, and can lead to acute poisoning among farmers, manifesting as symptoms like skin itching, rashes, and allergic conditions. The prevalence in India, approximately 40% of the total cultivated area is treated with pesticides [24].

Respiratory Distress

Respiratory disorders such as cough, wheezing, rhinitis, obstructive cardiovascular disorders, and tachycardia are frequently observed among agricultural workers. The prevalence of these disorders in India is approximately 3.59%, and similar trends are seen across the globe [18].

Lack of healthcare facilities for agricultural labor

In the Indian rural structure, agricultural workers constitute the most neglected class [24]. Assessing farmers' awareness and practices of safe toxic substance use is crucial for developing effective educational and policy strategies to minimize health and environmental risks linked with toxic substances [13]. Worldwide, most cultivation security technique programs are framed for individuals aged five to 12 years [25]. Training, encompassing hazard recognition, safe work practices, proper utilization of personal protective equipment, and understanding emergency procedures and preventive measures, emerges as a crucial intervention in mitigating farmers' pesticide exposure [12]. Although protective factors are not fully understood, existing evidence indicates that financial stability, social support, and a sense of belonging contribute positively to farmers' mental health [23]. It is crucial to prioritize the enforcement of current pesticide regulations and standards at both retail and farm levels through rigorous surveillance and monitoring efforts [8]. Furthermore, there is a need for health encouragement scheme activities to amplify the influence of organizational and psychological factors in the working area. Implementing health promotion programs focused on agriculture labor mental health could serve as other methods to enhance their overall quality of life [21].

Role of government initiatives 

According to the National Institutes of Health, occupational health is an ongoing endeavor to safeguard and advance workers' highest level of mental, social, and physical well-being across all professions [26]. Despite having numerous laws and schemes for worker welfare in India, there is insufficient emphasis on the health aspects of female agricultural workers [26]. The country conducted its first study assessing the incidence of employment discloses to numerous toxic agents, aiding in the ambulatory care provided by a reference center for agriculture labor health [27]. Improving the comprehensibility of labels on pesticides and agricultural products, coupled with motivating agricultural workers to seek formal education and access basic health services, will empower them with the necessary knowledge and resources to implement effective health protection measures for themselves and their families [18]. Most of the workers wore hats, with the practice of wide-brimmed hats being particularly noteworthy for minimizing sun exposure and the risk of skin cancer. The findings are crucial for identifying and establishing essential care actions that could enhance overall care, avoidance of pesticides, and a better standard of living for this agriculture framer [22,28]. Governments must recognize their responsibility as guardians of public health. They should establish laws and public policies with objectives beyond just enhancing land productivity, and ensuring the safeguarding of workers against potential occupational hazards [18]. The government scheme is known as the Pradhan Mantri Shram Yogi Maandhan Yojana is designed for the elder person safety and social security of disorganized agriculture laborers [29].

Selection of appropriate personal protective equipment

Using PPE can help minimize exposure to hazards found on farms. It is important to take preventative measures to ensure your safety [30]. Farm workers should follow protective measures to safeguard themselves from contamination while handling pesticides [15]. The farmers wore PPE such as body suits, goggles, face shields, masks, earmuffs, and plugs, which are essential to decrease the risk of acute injuries and long-term health impacts [30]. During the survey of farming activities, it was found that 100% of respondents did not use PPE completely. Boots, gloves, glasses, trousers, and full-sleeved clothes are not worn fully by them in every farming activity. Typically, only one or two types of PPE are worn by the farmers. It is noted that farm laborers didn’t utilize full PPE because it is considered to be a hindrance in their work and movement [31]. Aprons and boots were frequently utilized. After spraying, clothes were changed by the farmers. It seems that female farmers in developing countries face vulnerability [17]. Farmers generally have adequate knowledge of how pesticides enter the body, how to maintain and store pesticides, and how to protect themselves from them ​​​​[32]. Due to a limited understanding of the dangerous impacts of poisons on human health and financial limitations, the majority of farmers did not utilize PPE [33].

Table 1 shows a summary of the included studies.

**Table 1:** List of included studies in the review.

Sr. No.	Author Name	Year	Type of article	Finding
1	Tabibi R et al. [1].	2018	Original Article	Occupational health programs for farmers aim to enhance the health of those working in agriculture.
2	Wagoner RS et al. [3].	2020	Original Article	This project underscores the necessity of specific actions to enhance hydration and mitigate heat stress in the area.
3	Marcelino AF et al. [6].	2019	Original Article	Intensive training and concerted efforts are essential to raise awareness of safety practices and shift attitudes among farm workers, aiming to prevent harmful environmental and anthropogenic impacts.
4	Damalas CA et al. [10].	2017	Original Article	The key discovery of this study underscores the beneficial impact of safety training on safety management.
5	Asaaga FA et al. [11].	2021	Mixed method study	Highlighting the significance of tailoring disease information and interventions to specific contexts and implementing participatory approaches that adequately consider social determinants of health are crucial in enhancing households' ability to adapt to Kyasanur Forest Disease and other neglected endemic zoonoses.
6	Walton AL et al. [12].	2019	Observational study	There is a need for specific strategies aimed at enhancing washing behaviors in the field.
7	Jallow MFA et al. [13].	2017	Original Article	Approximately 82% of farmers reported experiencing at least one symptom indicative of acute pesticide poisoning.
8	Migheli M et al. [14].	2021	Journal Article	Proposed public interventions to address the issue include promoting integrated pest management techniques, which could prove beneficial.
9	Gaber S et al. [15].	2012	Cross Sectional study	School education was related to higher levels of knowledge and behaviors regarding the agriculture.
10	Röösli M et al. [16].	2022	Review Article	Assessing the effectiveness of interventions aimed at reducing exposure, promoting behavioral changes, raising awareness, implementing policies, and enforcing regulations is crucial.
11	Ebenso B et al. [8].	2022	Cross sectional study	Proposed public interventions to address the issue include promoting integrated pest management techniques, which could prove beneficial.
12	Rahaman MM et al. [5].	2018	Observational study	This effort could foster sustainable agricultural practices and contribute to safeguarding Bangladesh's
13	Arcury TA et al. [34].	2019	Qualitative study	Their training focuses more on task completion rather than ensuring task completion safely.
14	Chen Y et al. [9].	2022	Original Article	The analysis of mediating effects reveals that perceptions of health impact, self-efficacy, and adaptive costs partially mediate the influence of characteristics on adaptive behaviors.
15	Shin DS et al. [22].	2022	Original Article	The level of well-being significantly influenced musculoskeletal pains.
16	Meenakshi JR et al. [27].	2020	Original Article	Occupational health hazards significantly affect both the physical and mental well-being of female workers.
17	Lari S et al. [19].	2023	Original Article	Utilizing PPE during pesticide applications and other agricultural tasks is crucial for mitigating adverse health effects associated with pesticides.

Limitations

The agriculture workers have poor or low-income status so that workers are not afforded the complete set of PPE [35]. The agricultural workers have inadequate knowledge and training about the use of pesticides, so they are continuously exposed to multiple chemicals. Also, rural areas, where agricultural work is prevalent, often have limited access to healthcare facilities, making it difficult for workers to seek medical attention when needed [18]. 

## Conclusions

Overall agricultural workers face various health hazards such as respiratory problems and dermatological issues. Environmental pollution is increased by the misuse of pesticides and the risk of health-related issues the farmers face. The agricultural workers do not have proper knowledge and adequate training to practice PPE and are more at risk of exposure to pesticides commonly used in the agricultural sector. The selection of appropriate PPE is essential to reduce all health-related problems that agricultural workers face. Farmers often use improper PPEs due to perceived interference with their activities. Therefore, safety training and interventions are crucial, and priority should be given to enforcing existing pesticide laws. Agricultural workers addressing these challenges requires improved education regarding agricultural hazards, access to training about proper PPE kit use, effective enforcement of safety regulations gaps in the knowledge, and serious risks to agricultural workers.
